# A simple and quantitative ultrasonographic screening method for fetal Sylvian fissure abnormalities

**DOI:** 10.1186/s40001-025-02708-y

**Published:** 2025-06-18

**Authors:** Cuixia Guo, Lijuan Sun, Yan Liu, Tiejuan Zhang, Li Wang, Yuting Liang, Jingjing Wang, Qingqing Wu

**Affiliations:** 1 Department of Ultrasound, Beijing Obstetrics and Gynecology Hospital, Capital Medical University, Beijing Maternal and Child Health Care Hospital, Beijing, 100026 China; 2 Department of Prenatal Diagnostic Center, Beijing Obstetrics and Gynecology Hospital, Capital Medical University, Beijing Maternal and Child Health Care Hospital, Beijing, China; 3 Department of Radiology, Beijing Obstetrics and Gynecology Hospital, Capital Medical University, Beijing Maternal and Child Health Care Hospital, Beijing, China; 4 Clinical Trial Institution Office, Beijing Obstetrics and Gynecology Hospital, Capital Medical University, Beijing Maternal and Child Health Care Hospital, Beijing, China

**Keywords:** Sylvian fissure, Fetus, Ultrasonography, Malformation of cortical development, Genetic tests

## Abstract

**Objective:**

To explore a simple quantitative method for detecting fetal abnormal Sylvian fissure (SF) on ultrasonographic screening and to investigate its value in fetuses with SF abnormalities.

**Methods:**

As the control group, 128 patients were examined prospectively by 2D ultrasound from 22 to 31 gestational weeks. We measured the depth of the SF (SFD) and width of the SF (SFW) and defined a new parameter, namely, the SF ratio (SFR), as the ratio of the SFW to the SFD. Reference equations were constructed for the SF parameters and gestational age (GA). Thirty-eight fetuses with SF abnormalities were included in the study group.

**Results:**

In total, 310 ultrasound examinations were performed on 128 patients in the control group. The plateau-like insula was always present, and the SFD, SFW, and SFR increased with increasing gestational weeks between 22 and 31 gestational weeks. SFD (mm) = −1.48GA^2^−0.02GA+16.81 (*R*^*2*^ = 0.803). SFW (mm) = 5.62GA^2^−0.08GA–79.34 (*R*^*2*^ = 0.880). SFR = 0.53GA^2^−0.01GA-6.55 (*R*^*2*^ = 0.619). The SFR was >0.7 from 22 to 24 gestational weeks and >1 after 25 gestational weeks in the control group. In the study group, the SFD, SFW, and SFR values were below the 5th percentile of the respective normal ranges in 26/38 (68.4%), 38/38 (100%), and 38/38 (100%) patients with abnormal SF. There were significant differences in three parameters between the two groups (*P*<0.05). With respect to the final diagnosis, 30/38 (78.9%) fetuses had malformations of cortical development (MCD), and 8/38 (21.1%) fetuses had multiple CNS malformations.

**Conclusions:**

The SFR was >0.7 from 22-24 gestational weeks, and a value >1 after 25 weeks may serve as a simple parameter for detecting abnormal SF. An abnormal SF tends to be an indicator of CNS anomalies, especially MCD.

## Introduction

The Sylvian fissure (SF) is the most obvious landmark of the lateral brain surface [1]. The formation of the SF, also called ‘operculization’, involves a dynamic process with a precise timetable during pregnancy [2]. It is a complex space bordered by asymmetric cortical growth between the frontoparietal and temporal opercula enfolding the insular cortex. The maturation of the SF is a major landmark in cortical development. SF abnormalities can indicate cortical dysplasia or neuronal migration disorders [3] and can be related to extracortical cerebral abnormalities [4], some chromosomal anomalies or single-gene syndromes [5]; these conditions are often severe and have a poor prognosis. Accurate prenatal assessment of SF is crucial for distinguishing normal values from abnormal ones.

The assessment of normal operculization on ultrasound has been described previously. The SF can be identified as early as 12 gestational weeks on prenatal imaging, and its appearance changes markedly from shallow depressions to deep sulci during pregnancy [6, 7]. Prenatally, SF is evaluated mainly in the axial plane during the second and third trimesters of pregnancy. Some researchers have focused on biometric measurements to evaluate the SF. It is simple and easy to measure only the depth of the SF [7–10], but the measurement of only one parameter is obviously not sufficiently comprehensive for in the evaluation of SF. Some authors have reported the use of several parameters, such as those used on 2D [11] or 3D ultrasound [12, 13], to evaluate SF, but these methods are complicated and time-consuming. The respect to the planes, the coronal planes [14, 15] are not easy to obtain in daily practice. Zeng et al. [16] proposed the SF plateau angle to evaluate the SF plateau in axial planes at 23–28 weeks of gestational age (GA); its value is limited to a narrow GA range and needs to be tested on more abnormal cases of SF abnormalities. In one study, the morphological features of SF were described by means of cortical grading [17] in the axial plane; however, this method is too complex for clinical practice. Liao et al. subjectively categorized SF abnormalities into six groups [5]; for inexperienced doctors, it is not easy to define them as normal or abnormal subjectively during screening, especially when a narrow plateau-like insula is present. Therefore, further studies to simplify the evaluation of SF abnormalities, especially objective methods for screening, are lacking.

Here, we aimed to explore an objective and simple method for fetal SF screening and evaluate its value in fetuses with SF abnormalities.

## Methods

**Fig. 1:**
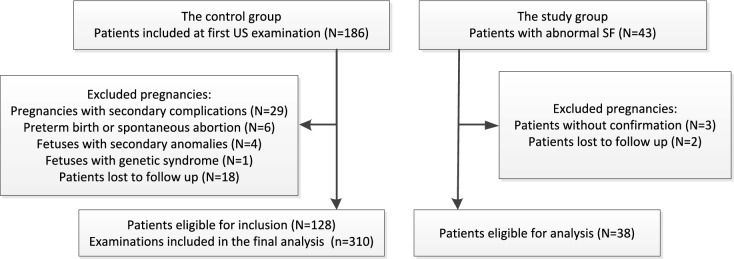
The flowchart of summarizing inclusion of pregnancies in the two groups

The study population included two groups of patients who attended our center. In the control group, we prospectively enrolled women with singleton pregnancies from December 2021 to November 2023. For each patient, we carried out 1–3 ultrasound examinations from 22–31 gestational weeks. The inclusion criteria were as follows: (1) women aged between 18 and 38 years; (2) uncomplicated pregnancies with low risk factors pregnancies; (3) fetal size appropriate for GA, which was determined by the crown–rump length (CRL) measured in the first trimester; (4) no fetal structural or genetic anomalies throughout the pregnancy; and (5) no malformations at birth. The exclusion criteria were as follows: (1) pregnancies with secondary complications, preterm birth or spontaneous abortion; (2) fetuses with secondary anomalies or genetic syndrome; and (3) patients lost to follow-up. Birth and newborn records were collected from a medical electronic system. In the study group, we selected fetuses (10 retrospectively, 28 prospectively) that were confirmed to have a Sylvian fissure abnormality on fetal MRI from 2019-2024. A flowchart of the inclusion of the two groups is presented in Fig. 1.

All examinations were performed by two operators (CX Guo and LJ Sun) with a Samsung HERA WS80 A/W10 (Samsung, Korean), Voluson E8/E10 ultrasound system (GE Healthcare) or Philips EPIQ 7 (Philips Ultrasound, Inc.) with transabdominal and transvaginal transducers. After routine screening, we carried out transabdominal neurosonography in all patients, and a transvaginal approach was used when the fetuses were in the cephalic position.

**Fig. 2:**
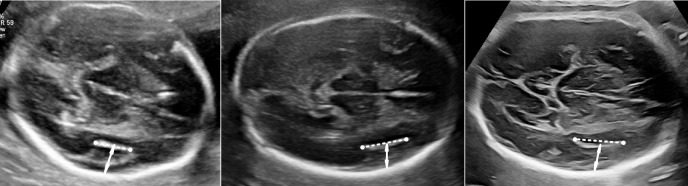
The measurements of width (dashed line) and depth (arrow) of SF at 23 weeks (**a**), 26 weeks (**b**), and 30 weeks (**c**)

The measurements in the two groups were all made by ultrasonography. The measurements of SF in the distal hemispheres were performed in the transthalamic axial plane of the brain with visualization of columns of the fornix to define the plane precisely [18]. First, we identified the midpoint of the SF plateau. The plateau of the SF was defined as the formation of angular margins at both the anterior site of the circular SF in the frontal lobe and the posterior site in the temporal lobe. The depth of the SF (SFD) was measured from the midpoint of the SF plateau (including the hyperechogenic border of the insula) to the inner surface of the parietal bone, perpendicular to the falx cerebri. The width of the SF (SFW) was measured as the distance between two end points of the SF plateau resulting from the peri-insular sulci in the frontal and temporal lobes (Fig. 2). We defined a new parameter, namely, the SF ratio (SFR), which is calculated as the ratio of the SFW to the SFD. In patients without the formation of a plateau-like insula, the SFD was measured from the peak of the SF to the inner surface of the parietal bone, perpendicular to the falx cerebri. All the measurements were performed by one observer (CX Guo). To assess the intraobserver and interobserver reproducibility of the measurements, stored images from 30 patients were selected randomly, and a second operator (LJ Sun) performed the measurements and a second time by CX Guo, who were both blinded to the previous measurements.

The results of fetal magnetic resonance imaging (MRI) and genetic tests were collected as the confirmation of abnormal SF. The final diagnosis was based on the results of genetic tests first and then on fetal MRI. Genetic tests, including karyotyping, chromosomal microarray, and trio-whole exome sequencing (trio-WES), were performed by means of amniocentesis or the use of abortion tissues. All patients signed written consent forms, and the study was approved by the institutional ethics committee of Beijing Obstetrics and Gynecology Hospital, Capital Medical University (No. 2022-KY-034-02).

## Statistical analysis

The data were analyzed with the statistical software SPSS version 24.0 (SPSS, Chicago, Illinois, USA). Continuous variables are presented as the mean ± standard deviation (SD) or P50 (P5, P95). Categorical variables are presented as frequencies (percentages). Correlations between GA and SFD, SFW, or SFR were evaluated by means of the Spearman rank correlation coefficient. Polynomial regression analysis was used to obtain the best fitted regression curves. The best fit function was selected according to the highest adjusted R^2^ coefficient. The intra- and inter-observer repeatability of the measurements were assessed by means of the interclass correlation coefficient (ICC). An ICC above 0.75 indicated good agreement. Nonparametric tests were used to analyze the differences in clinical characteristics and ultrasonic measurements between the two groups. A *p* value< 0.05 was considered statistically significant.

## Results

In the control group, a total of 310 scans of 128 fetuses were performed between 22 and 31 weeks of gestation. Thirty-eight fetuses with an abnormal SF were included in the study group. All pregnancies were singletons. The demographic characteristics of the two groups are presented in Table 1. Except for BMI, there were no significant differences in the clinical characteristics of the two groups. The patients with an abnormal SF seemed to have a higher BMI (*P*<0.05).

**Table 1:** Demographic characteristics and ultrasonographic measurements in the two groups

Characteristic	Value		*P* value
	Control group (*n* = 128)	Study group (*n* = 38)	
Maternal age (years)	31.8 ± 2.3	31.2 ± 5.4	.213
Maternal BMI (kg/m^2^)	21.4 ± 2.4	25.4 ± 4.4	.000
Parity			
Nulliparous	98 (76.6%)	24 (63.2%)	.142
Multipara	20 (23.4%)	14 (36.8%)	
*Baby gender*			
Male	69 (53.9%)	20(52.6%)	1.000
Female	59 (46.1%)	18 (47.4%)	
Gestational age (weeks)	25.7 ± 3.0	25.5 ± 2.4	.364
*Delivery mode*			
Natural	73 (57.0%)		
Cesarean	42 (32.8%)		
Forceps	13 (10.2%)		
Birth weight (g)	3365 ± 377		
Birth weeks	39.1±0.9		

In the control group, the plateau-like insula was present in all fetuses between 22 and 31 weeks of gestation. The shape of the plateau changed from a line at 22 weeks to a curve at 31 weeks. The ultrasound measurements, including SFD and SFW, increased with increasing GA. The best fit curve for the measurements was a polynomial second-order relationship with the GA. SFD (mm) = −1.48GA^2^−0.02GA+16.81 (*R*^*2*^ = 0.803), SFW (mm) = 5.62GA^2^−0.08GA–79.34 (*R*^*2*^ = 0.880). The value of SFR also increased with the advancing GA; SFR = 0.53GA^2^−0.01GA–6.55 (*R*^*2*^ = 0.619). The SFR was >0.7 for all fetuses between 22 and 24 gestational weeks and >1 after 25 gestational weeks. The measurements of the SFD, SFW, and SFR were recorded as P50 (P5, P95) according to the number of gestational weeks, as shown in Table 2.

**Table 2:** The measurements of SFD, SFW, and SFR according to gestational weeks in the control group

Gestational ages	P50 (P5, P95)		
	SFD (mm)	SFW (mm)	SFR
22w (n = 40)	8.8 (7.3–10.1)	8.1 (6.2–12.7)	0.94 (0.75–1.30)
23w (n = 59)	8.9 (7.6–10.1)	8.9 (6.4–11.3)	1.00 (0.77–1.23)
24w (n = 13)	10.2 (9.7–11.0)	12.1 (7.0–13.5)	1.12 (0.72–1.33)
25w (n = 32)	10.6 (8.9–12.3)	14.4 (11.2–16.0)	1.28 (1.10–1.51)
26w (n = 38)	11.3 (9.1–13.0)	14.8 (12.7–18.2)	1.33 (1.10–1.86)
27w (n = 16)	11.9 (10.6–13.1)	16.8 (14.5–18.7)	1.38 (1.20–1.60)
28w (n = 10)	12.6 (10.1–14.8)	17.6 (14.6–18.7)	1.38 (1.26–1.45)
29w (n = 21)	13.4 (11.9–15.9)	18.6 (15.5–20.7)	1.33 (1.15–1.74)
30w (n = 62)	13.4 (11.5–14.6)	20.5 (16.5–23.4)	1.52 (1.20–1.78)
31w (n = 19)	14.5 (13.2–15.6)	20.3 (17.8–23.4)	1.38 (1.17–1.68)

**Fig. 3:**
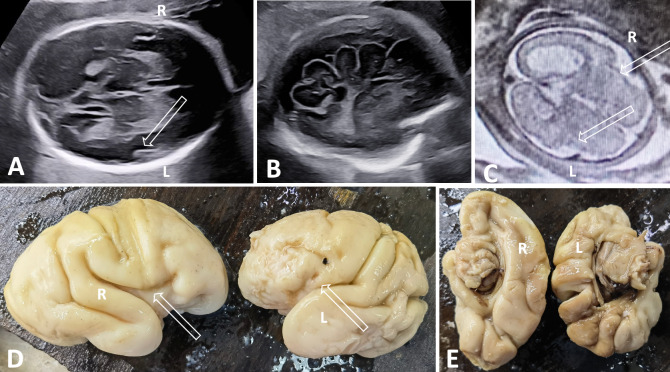
Patient 6, the 24.6-gestational-week-old fetus of a 34-year-old woman, had megalencephaly–polymicrogyria (L) and ventriculomegaly (R). **A** Axial plane showing irregular SF without a plateau-like insula (hollow arrow). **B** Parasagittal plane showing abnormal sulci in the left hemisphere with a seesaw appearance. **C** Axial MR image showing hemisphere asymmetry, abnormal SF (hollow arrow, L) and ventriculomegaly (R). **D** Comparison of lateral surfaces of the brain by postmortem examination showing abnormal SF (hollow arrow, L) and polymicrogyria in the frontal lobe (L, arrows). **E** Comparison of the medial surfaces of the brain by postmortem examination revealed that the left hemisphere was smaller with abnormal sulci

**Fig. 4:**
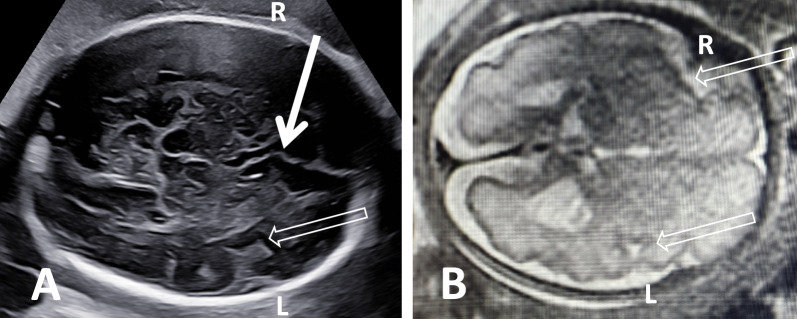
Patient 30, the 29.3-week-old fetus of a 30-year-old woman, had bilateral abnormal SF. **A** Axial plane showing an abnormal SF (hollow arrow) in the distal hemisphere; note the distortion of the anterior midline (arrow). **B** MR image showing the asymmetric sulci on the lateral surface of the brain; note the narrow-plateau SF in the left hemisphere and the nonplateau SF in the right hemisphere (hollow arrows). WES-Trio revealed a variant in the *TUBB3* gene, which may cause complex cortical dysplasia with other brain malformations

**Fig. 5:**
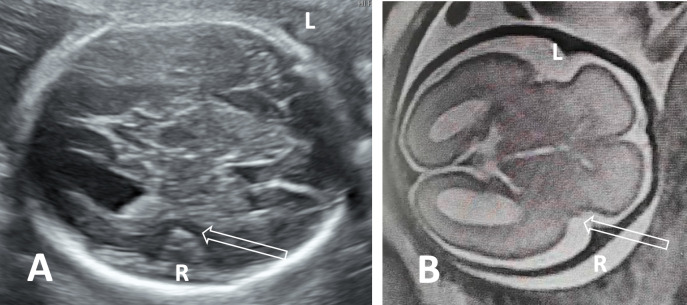
Patient 31, the 27.1-gestational-week-old fetus of a 29-year-old woman, had a narrow plateau-like insula in the right hemisphere. **A** Axial plane shows the comparison of the abnormal SF (hollow arrow, R) and the normal SF (L); **B** axial plane by MRI shows similar characteristics of SF (hollow arrow, R) with ultrasound. WES-Trio revealing a variant in the *CYNC1HI* gene that may cause mental delay

**Fig. 6:**
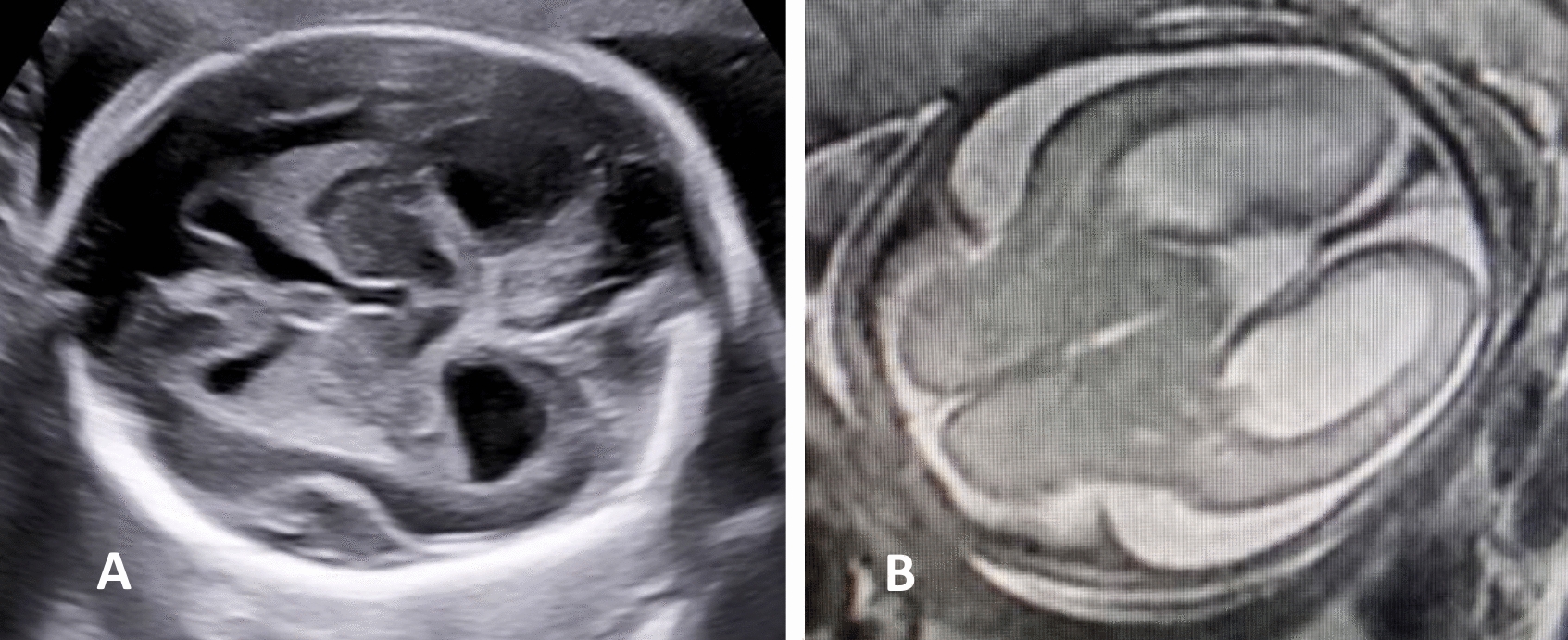
Patient 38, the fetus of a 32-year-old woman, had bilateral delayed SF and hydrocephalus. **A** Axial plane at 24.9 weeks by ultrasound showing delayed and trapezoid-shaped SF and bilateral hydrocephalus. **B** Axial plane at 25 weeks by MRI showing bilateral delayed SF and bilateral hydrocephalus

In the study group, fetal MRI was performed in all patients. SF was abnormal bilaterally in 29 (29/38, 76.3%) patients and unilaterally in 9 (9/38, 23.7%) patients. In most cases (Cases 1–29, Fig. 3), the plateau-like insula did not form. Interestingly, one patient (Patient 30, Fig. 4) did not have a plateau-like insula on the right and had a narrow plateau-like insula on the left. The plateau-like insula was present in 9 patients (Cases 30–38), including 7 patients with a narrow plateau-like insula (Cases 30–36, Fig. 5) and 2 patients with delayed SF (Cases 37–38, Fig. 6). The SFD measurements were below the 5 th percentile of the normal value in 25 of 38 (65.8%) patients and above the 95 th percentile of the normal range in 2/38 (5.3%) patients (Cases 31 and 35). The SFW and SFR were below the 5 th percentile of the normal reference values in 38/38 (100%) patients. Although most cases (Cases 1–29, 76.3%) without a plateau-like insula could be identified subjectively, the measurements of SFW and SFR provided value at least in approximately 23.7% cases (Cases 30–38).

The final diagnosis included 30 (78.9%) cases of malformations of cortical development (MCD) (including microcephaly (*n* = 7)), 5 (13.2%) cases of delayed sulci, and 3 (16.7%) cases of other CNS anomalies. Among the 29 patients without the formation of a plateau-like insula, 25 (86.2%) had MCD (including microcephaly (*n* = 7)), 3 (10.3%) had delayed sulci, and 1 (3.4%) had asymmetric sulci. Among the 9 patients with a plateau-like insula, 5 (55.6%) fetuses had MCD, 2 (22.2%) had delayed sulci, and 2 (22.2%) had other CNS anomalies. All (38/38, 100%) cases were associated with other anomalies in the CNS, including ventriculomegaly (*n* = 21, 12 unilateral)/hydrocephalus (*n* = 8, 5 unilateral) (29/38, 76.3%), callosal anomalies (*n* = 13, 34.2%), cerebellar/vermian hypoplasia (*n* = 12, 31.6%), a small head circumference (*n* = 9, 26.7%), and a distorted midline (*n* = 5, 13.2%). Associated anomalies in the extra-CNS were detected in only 10 (10/38, 26.3%) of the cases. In the study group, genetic tests were carried out on 20 patients. In total, 13/20 (65%) cases had positive results, including copy number variations of microdeletions at 17p13.3 (case 13, Miller–Dieker syndrome) and 6q22 (case 36) and pathogenic or likely pathogenic variants in *TUBB3*, *TUBB*, *CYNC1HI*, *CCND2*, *IQSEC2, DPYSL5, RTTN, KIF11*, *OFD1,* and *TUBG1*. With regard to the outcomes, 35 patients terminated their pregnancies, and 3 patients (1 case of multiple CNS anomalies, 1 case of pons hypoplasia, and 1 case of tethered spinal cord syndrome) delivered at term. The measurements and characteristics of the SF, imaging findings, genetic results, and outcomes of the fetuses in the study group are presented in Tables 3 and 4.

**Table 3:** The SF characteristics, imaging findings, genetic tests, and outcomes of 29 patients without a plateau-like insula in the study group

Case	GA at diagnosis	SF laterality	SF measurements			CNS findings	Extra-CNS findings	Final diagnosis	Genetic testing/underlying disease	Outcomes
			SFD	SFW	SFR					
1	22	Bilateral	4.1	0	0	Hemimegalencephaly, asymmetric hemispheres, distorted midline, ventriculomegaly (R), CH	–	Hemimegalencephaly	Unavailable	TOP
2	26	Bilateral	5.5	0	0	FGR, delayed sulci, enlarged subarachnoid space	Cutaneous dropsy, absence of nasal bone	Polymicrogyria	*RTTN* (+); polymicrogyria with seizures	TOP
3	23	Bilateral	5.7	0	0	Hemimegalencephaly, schizencephaly, hydrocephalus (R), short CC, rhomboencephalosynapsis (RES)	–	Hemimegalencephaly, schizencephaly	*TUBG1* (+); complex cortical dysplasia with other brain malformations	TOP
4	25	Bilateral	11.5	0	0	Megalencephaly–polymicrogyria; hydrocephalus (L), ventriculomegaly (R), enlarged ganglionic eminence	Thoracic vertebra dysplasia, polydactyly	Megalencephaly–polymicrogyria	*CCND2* (+); megalencephaly–polymicrogyria–polydactyly–hydrocephalus syndrome, type 3	TOP
5	25	Bilateral	8.2	0	0	Distorted midline, ventriculomegaly (L)	–	Cortical dysplasia	*TUBB* (+), complex cortical dysplasia with other brain malformations, type 6	TOP
6	24	Unilateral (L)	7.0	0	0	Megalencephaly–polymicrogyria, asymmetry hemisphere, callosal hypoplasia, ventriculomegaly (B), midline cyst	–	Megalencephaly–polymicrogyria	Unavailable	TOP
7	22	Bilateral	6.6	0	0	Pachygyria, ACC, ventriculomegaly (B), absent CSP	–	Pachygyria	Karyotype (−), CNVs (−), Trio-WES (−)	TOP
8	26	Unilateral (R)	6.5	0	0	Schizencephaly, ACC, absent CSP, MCM, VH, midline arachnoid cyst	–	Schizencephaly	*OFD1*(+); oral–facial–digital syndrome type 1	TOP
9	24	Bilateral	9.3	0	0	Asymmetric sulci, ventriculomegaly (B), ACC, absent CSP	–	Cortical dysplasia	Unavailable	TOP
10	25	Bilateral	11.4	0	0	Ventriculomegaly (B), ACC, absent CSP	–	MCD	*DPYSL5* (+), Ritscher–Schinzel syndrome, type 4	TOP
11	25	Unilateral (L)	9.2	0	0	Ventriculomegaly (L), asymmetric sulci	PLSVC , right subclavian artery vagus, SUA	Cortical dysplasia	Unavailable	TOP
12	23	Bilateral	5.1	0	0	Ventriculomegaly (B), ACC, VH, MCD	Polyhydramnios	MCD	Unavailable	TOP
13	28	Bilateral	11.5	0	0	Lissencephaly, ventriculomegaly (B), delayed sulci	Polyhydramnios, small stomach, meso-position liver	lissencephaly	Del(17)(p13.3p13.3), 1.78 Mb; Miller–Dieker syndrome	TOP
14	29	Bilateral	10.3	0	0	BPD &HC<1%, CH, VH, ACC, MCM	Unilateral pyelectasis	Microcephaly	Unavailable	TOP
15	22	Bilateral	5.0	0	0	BPD &HC<1%, delayed sulci	–	Microcephaly	Unavailable	TOP
16	25	Bilateral	0	0	0	BPD &HC<1%, delayed sulci, small frontal lobe, enlarged subarachnoid space	–	Microcephaly	Unavailable	TOP
17	31	Bilateral	5.9	0	0	Microcephaly, BPD &HC<1%, short CC, CH	–	Microcephaly	Unavailable	TOP
18	27	Bilateral	6.1	0	0	Microcephaly, BPD &HC<1%, callosal hypoplasia	–	Microcephaly	Unavailable	TOP
19	30	Bilateral	9.4	0	0	Ventriculomegaly (L), BPD &HC<1%, CH, OSB, meningocele	–	Microcephaly	Unavailable	TOP
20	25	Bilateral	7.9	0	0	Microcephaly, BPD and HC<1%, delayed sulci	Absent ductus venosus	Microcephaly	*KIF11* (+); microcephaly with or without chorioretinopathy, lymphedema, or mental retardation	TOP
21	26	Bilateral	8.1	0	0	Microcephaly, BPD and HC<1%, smooth brain, enlarged subarachnoid space	–	Microcephaly, lissencephaly	Unavailable	TOP
22	22	Bilateral	5.1	0	0	Ventriculomegaly (B), distorted midline, pACC, VH	–	Cortical dysplasia	Karyotype (−), CNVs (−), WES-Trio (−)	TOP
23	23	Bilateral	9.8	0	0	HC<3%, asymmetric sulci, Rhomboencephalosynapsis (RES)	–	RES, cortical dysplasia	Karyotype (−), CNVs (−), WES-Trio (−)	TOP
24	24	Bilateral	3.2	0	0	Hydrocephalus (B), ACC, delayed sulci	pleural effusion	Delayed sulci	Unavailable	TOP
25	24	Bilateral	6.8	0	0	Hydrocephalus (B), ACC, delayed sulci	–	Delayed sulci	Unavailable	TOP
26	23	Bilateral	5.2	0	0	Hydrocephalus (B), dilated 3 th ventricle, delayed sulci, thin parenchyma	–	Cortical dysplasia	Unavailable	TOP
27	24	Bilateral	8.2	0	0	Hydrocephalus (R), ventriculomegaly (L), small TCD	–	Delayed sulci	Unavailable	TOP
28	23	Bilateral	7.1	0	0	Ventriculomegaly (L), distorted midline	–	Cortical dysplasia	*IQSEC2* (+), Rett syndrome	TOP
29	22	Bilateral	8.2	0	0	Ventriculomegaly (B), asymmetric sulci	Double aortic arch, abnormal toes	asymmetric sulci	Karyotype (−), CNVs (−), WES-Trio (−)	TOP

*SF* Sylvian fissure; *CH* cerebellar hypoplasia; *VH* vermian hypoplasia; *MCD* malformation of cortical development; *OSB* open spina bifida; *RES* rhomboencephalosynapsis; *TCD* transverse cerebellar diameter; *CNVs* copy number variations; *WES* whole exome sequencing; *TOP* termination of pregnancy; *CSP* cavity of septum pellucidum; *MCM* mega cisterna magna; *PLSVC* persistent left superior vena cava; *SUA* single umbilical artery; *BPD* biparietal diameter; *HC* head circumference; *TCD* transverse cerebellar diameter; *FL* femur length; *CC* corpus callosum; *ACC* agenesis of corpus callosum; *FGR* fetal growth restriction; *L* left; *R* right; *B* bilateral

**Table 4:** The SF characteristics, imaging findings, genetic tests, and outcomes of 9 patients with a plateau-like insula in the study group

Case	GA at diagnosis	SF laterality	SF measurements			CNS findings	Extra-CNS findings	MRI diagnosis of MCD	Genetic testing/underlying disease	Outcomes
			SFD	SFW	SFR					
30	29	Bilateral, no-plateau (right), narrow-plateau (left)	11.9	8.1	0.68	Distorted midline, delayed and abnormal sulci, narrow CSP, mild ventriculomegaly (L), MCM	–	MCD	*TUBB3* (+); cortical dysplasia, complex, with other brain malformations 1	TOP
31	27	Right, narrow-plateau	13.3	6.1	0.46	Mild ventriculomegaly (R), asymmetric and abnormal SF (R), abnormal superior temporal sulcus (R)	–	Cortical dysplasia	*CYNC1HI* (+); mental retardation, autosomal dominant 13; OMIM:614563	TOP
32	26	Left, narrow-plateau	6.7	6.2	0.93	Mild ventriculomegaly (L), asymmetric and abnormal SF (L), absent superior temporal sulcus (L)	–	Cortical dysplasia	*TUBB3* (+); cortical dysplasia, complex, with other brain malformations 1	TOP
33	25	Right, narrow-plateau	9.9	6.7	0.68	Asymmetric sulci, abnormal SF (R), tethered spinal cord syndrome	–	Tethered spinal cord syndrome	Karyotype (−), CNVs (−), WES-Trio (−)	Newborn, NND
34	25	Left, narrow-plateau	9.7	5.3	0.55	Mild ventriculomegaly (L), asymmetric sulci, abnormal SF (L), pons hypoplasia	–	Pons hypoplasia	Unavailable	Liveborn, NND at 1.5 yr
35	30	Left, narrow-plateau	16.3	12.5	0.75	Hydrocephalus (L), asymmetric and abnormal SF (L), irregular insula (L)	–	Megalencephaly–polymicrogyria	Unavailable	TOP
36	24	Left, narrow-plateau	8.8	5.0	0.57	Mild ventriculomegaly (L), delayed and asymmetric sulci, small TCD	–	MCD	SNP array revealed deletion in 6q27 (2.2Mb)	TOP
37	26	Bilateral delayed	12.3	12.5	1.02	Mild ventriculomegaly (B), CH, MCM, delayed sulci, enlarged subarachnoid space	Small FL	Delayed sulci	Karyotype (−), CNVs (−), WES-Trio (−)	TOP
38	24	Bilateral delayed	10.1	6.2	0.61	Hydrocephalus (B), delayed sulci, midline cyst, callosal hypoplasia, enlarged subarachnoid space	–	Delayed sulci	Karyotype (−), CNVs (−), WES-Trio (−)	Liveborn, NND at 2.5 yr

*SF* Sylvian fissure; *CH* cerebellar hypoplasia; *VH* vermian hypoplasia; *MCD* malformation of cortical development; *OSB* open spina bifida; *RES* rhomboencephalosynapsis; *TCD* transverse cerebellar diameter; *CNVs* copy number variations; *WES* whole exome sequencing; *TOP* termination of pregnancy; *NND* normal neurodevelopmental development; *CSP* cavity of septum pellucidum; *MCM* mega cisterna magna; *PLSVC* persistent left superior vena cava; *SUA* single umbilical artery; *BPD* biparietal diameter; *HC* head circumference; *TCD* transverse cerebellar diameter; *FL* femur length; *CC* corpus callosum; *ACC* agenesis of corpus callosum; *FGR* fetal growth restriction; *L* left; *R* right; *B* bilateral

The intra- and inter-observer agreement was excellent, with an ICC of 0.991 (95% CI, 0.981–0.996) and 0.984 (95% CI, 0.968–0.993), respectively, for SFD, and an ICC of 0.976 (95% CI, 0.950–0.989) and 0.957 (95% CI, 0.912–0.979), respectively, for SFW.

## Discussion

Our findings revealed that the SFD and SFW increased with increasing GA between 22 and 31 weeks of gestation and that the SFW was more effective than the SFD for identifying SF abnormalities. We propose a new parameter, namely, the SFR, which also increases with GA from 22 to 31 gestational weeks. Its value is >0.7 from 22 to 24 gestational weeks and >1 after 25 gestational weeks in the control group. The SFR can serve as an objective and simple parameter for SF screening between 22 and 31 gestational weeks.

The SFW and, especially, the SFR may serve as potential indicators for SF screening. In the study group, the value of each of the two parameters was below the 5^th^ percentile of the respective normal range. The SF ratio has rarely been mentioned in the literature, the SF ratio defined in only one study; however, this ratio reflects the changes in SF with depth [8], and its value has not been tested in abnormal cases. In our study, the SFR reflected the dynamic changes in the SF in both the lateral and anterior‒posterior directions, indicating that the SFR has greater potential value for the anatomical development of the SF. Chen et al reported the SFW in normal fetuses [12], and the growth pattern aligned with GA in our study; however, the authors did not apply the SFW in cases of SF abnormalities. Here, we tested the value of using the SFW and SFR for the first time in cases of SF abnormalities SF. Although, as shown in our study, most SF abnormalities can be detected subjectively, and detection of the SF with a narrow plateau-like insula or more subtle anomalies is still a challenge for inexperienced examiners. In such cases, we recommend the SFR as a simple and quantitative parameter for SF screening.

Measurement of the SFD in the axial plane is simple, and this value is the most frequently used by researchers; however, its value is limited. In our study, the SFD values in 11 (28.9%) cases of abnormal SF were within the normal range. Several authors [8, 12, 19] have reported measurements of SFD in the axial plane via 2D ultrasound, but they did not apply this parameter to cases of abnormal SF. The SFD does not accurately reflect SF development. SF grows more rapidly in the anterior‒posterior direction than in the superior‒inferior and lateral directions [13]. Moreover, the measurement of the SFD usually includes the subarachnoid space, which increases the value of the SFD. In our study, 2 patients (Patients 31 and 35) had enlarged subarachnoid spaces, resulting in increased SFD values. Benign enlargement of the subarachnoid space has been reported in a prenatal case [20]. In one study, the SFD was measured by excluding the subarachnoid space around the SF in a mid-coronal plane, and the SFDs were reported to be strikingly different in cases of cortical abnormalities, microcephaly, or both [14]. However, the coronal plane is not easy to obtain in daily practice. Therefore, the value and measurement of the SFD need to be studied further.

Abnormal SF on prenatal imaging can be an imaging indicator of underlying MCD [3, 4]. SF can be recognized with a smooth margin in the first trimester on ultrasound [21]. After 17 weeks of gestation, the operculum takes on a plateau-like appearance with angularity, and the angle changes from obtuse to acute after 24.5 weeks [22]. Chen et al. classified opercular abnormalities by MRI into two categories: an underdeveloped operculum and a malformed operculum [23]. The underdeveloped operculum is usually associated with brain anomalies, multiple malformations, intrauterine infection, metabolic disease, a chromosomal anomaly or a neurogenetic syndrome [4, 20]. The malformed operculum is defined as an unformed or abnormally formed operculum; it is associated with lissencephaly and pachygyria, and the prognosis is poor [23]. Abnormal SF has been associated with MCD in 33.3%–95.5% of cases [1, 4, 5]. In the study group, most fetuses had MCD, which was consistent with the findings of previous studies. MCD can be unilateral, focal, or more extensive and may occur without abnormal operculization [24]. Prenatal evaluation of cortical development should not be limited to the SF.

In all cases of abnormal SF, the SF abnormality was associated with other CNS anomalies, as reported in a previous study [4]. However, associated extra-CNS anomalies are not common or nonspecific. Notably, ventriculomegaly/hydrocephalus, especially mild ventriculomegaly or asymmetric ventricles, was the most commonly associated CNS anomaly. Although delayed SF has been reported in fetuses with ventriculomegaly [25, 26], fetuses with isolated mild ventriculomegaly tend to have good outcomes. In our study, three patients (Patients 31, 32, and 34) had seemingly isolated ipsilateral mild ventriculomegaly; however, two of them had MCD, one had pons hypoplasia, and one had a unilateral abnormal SF with a narrow plateau-like insula. We recommend the routine evaluation of the SF during screening. A finding of abnormal SF should alert sonographers to the possibility of MCD or a severe cerebral anomaly and prompt a detailed neurosonography examination or referral to fetal brain MRI and/or amniocentesis for genetic testing.

Our study has several strengths. First, we proposed a new parameter, the SFR, to simplify the quantitative evaluation of the SF during screening. Second, we established the normal ranges of the SFD, SFW and SFR at 22–31 gestational weeks and reported that the SFW and SFR were more effective than the SFD in detecting abnormal SF. The limitations of this study were as follows. The main limitation is the limited number of patients, so further prospective studies with large sample sizes and multicenter measurements are needed. Moreover, owing to the poor visualization of ultrasonographic imaging in the proximal hemisphere, only the measurements in the distal hemisphere were included for analysis in the control group.

## Conclusion

In this study, we demonstrated that the evaluation of SF in the axial plane on transabdominal 2D ultrasound is feasible. The SFW and SFR were more effective than the SFD in detecting abnormal SF. The SFR may serve as a simple screening tool for detecting abnormal SF, with the value being>0.7 from 22 to 24 gestational weeks and >1 after 25 gestational weeks. Finally, SF abnormalities in fetuses tended to be an indicator of CNS anomalies, especially MCD.

## Data Availability

The data are available from the corresponding author upon reasonable request.
